# COVID-19 History Increases the Anxiety of Mothers with Children in Intensive Care during the Pandemic in Turkey

**DOI:** 10.3390/children9101448

**Published:** 2022-09-22

**Authors:** Eren Yildiz, Zuhal Koc Apaydin, Berna Alay, Zübeyde Dincer, Emrah Cigri

**Affiliations:** 1 Department of Pediatrics, Kastamonu University Faculty of Medicine, Kastamonu 37150, Turkey; 2 Department of Psychiatry, Karabük University Faculty of Medicine, Karabük 78000, Turkey; 3 Department of Pediatrics, Kastamonu Training and Research Hospital, Kastamonu 37100, Turkey

**Keywords:** COVID-19, mothers, anxiety, pediatric intensive care units

## Abstract

This study aimed to examine the mental status of mothers whose children were hospitalized during the COVID-19 pandemic, especially in places where risk factors are higher such as pediatric intensive care units, and to contribute to the development of psychological health policies, especially for these high-risk groups in epidemic situations. Method: This descriptive cross-sectional study was conducted between January 2021 and July 2021. The population of the study was mothers whose children were hospitalized in the pediatric intensive care unit during the study period. Data collection was carried out via a face-to-face interview method by experienced nurses working in pediatric clinics using a sociodemographic data form, the Beck Anxiety Inventory, the Beck Depression Inventory, the Fear of COVID-19 Inventory, and the Coronavirus Anxiety Inventory. Results: The median age of the participants was 33 (min: 21, max: 50). The Beck Anxiety score was affected by the child’s diagnosis, location, and history of COVID-19 (* P = 0.011, ** P = 0.018, and *** P = 0.002, respectively). Similarly, the Beck Depression score was affected by the child’s diagnosis and history of COVID-19 in a relative (* P = 0.034 and ** P = 0.037, respectively). The Coronavirus Anxiety score was affected by a history of COVID-19 in a relative and work status (* P = 0.040 and ** P = 0.005, respectively), while the Fear of COVID-19 score was not significantly affected by any variable. In a logistic regression model, previous COVID-19 experience was independently associated with anxiety; a history of COVID-19 increased the risk of developing anxiety by approximately 15 times (odds ratio: 14.915, 95% CI: 2.075–107.192). Conclusion: When children of mothers with a history of COVID-19 are hospitalized, special attention should be given to their mothers concerning psychological support and assistance.

## 1. Objectives

Natural disasters, emergencies, extreme stress, and conflict situations can increase the risk of mental health morbidity [1]. The emotional burden associated with serious and deadly diseases such as COVID-19 can affect vulnerable people, leading to increased stress reactions, depression, suicide, and even psychosis [2]. As a matter of fact, in the COVID-19 pandemic, a disaster with 245.373.039 confirmed cases and 4.979.421 global deaths as of October 2021 [3], psychological problems such as anxiety and depression have increased [4].

The physical, psychological, and cognitive development of children is affected by the mental health of their mothers. During the COVID-19 pandemic, women are psychologically more vulnerable than men, and people with children are at a higher risk of stress and anxiety than those without [5]. In addition, there has been an increase in mental health problems such as clinically related anxiety and depression in mothers during the pandemic [6,7,8,9].

However, these studies were generally conducted with pregnant women or mothers of newborns [1,6,8]. The number of studies investigating the psychology of mothers who have children outside of the newborn period in the pandemic is relatively low.

Additionally, during the COVID-19 pandemic anxiety has increased among mothers with children who need more care [10]. Sickness and hospitalization of children can cause sudden changes in the lives of family members. The increase in the care needs of the child, the financial difficulties that may arise due to the family’s health expenses, and the restrictions in the daily lives of the members of the family may cause the parents to experience anxiety [11]. The fear of infecting their children with the disease and the possible negative consequences of the disease in mothers of these children, the fear of having less social support due to isolation processes, and the fear of not being able to receive adequate health support due to the excessive burden on healthcare teams may further adversely affect the mothers’ psychological health [12,13].

Considering all these factors, it was thought that the psychological state of the mothers of the children hospitalized in the intensive care unit (ICU) during the pandemic should be investigated. Since intensive care units are categorized as neonatal and pediatric and the number of studies on newborn mothers is quite high compared to the other mothers, the study was carried out in the pediatric intensive care unit (PICU).

This study aimed to examine the mental status of mothers whose children were hospitalized during the COVID-19 pandemic, especially in places where risk factors are higher such as pediatric intensive care units, and to contribute to the development of psychological health policies, especially for these high-risk groups in epidemic situations.

## 2. Method

### 2.1. Study Design

This descriptive cross-sectional study was conducted between January 2021 and July 2021. The population of the study was mothers whose children were hospitalized in the Kastamonu Training and Research Hospital PICU during the study period. The study reporting was conducted per STROBE guidelines [14].

### 2.2. Ethics Statement

Ethical approval (numbered 2020-KAEK-143-18.01, dated 7 January 2021) was obtained from the Kastamonu University Clinical Research Ethics Committee.

### 2.3. Setting

The Kastamonu Training and Research Hospital pediatric department has a capacity of 60 beds, welcoming approximately 100,000 patients for outpatient treatment and 7000 inpatients every year. In addition, there are 5 pediatric and 12 neonatal ICU beds.

### 2.4. Participants

**Figure 1:**
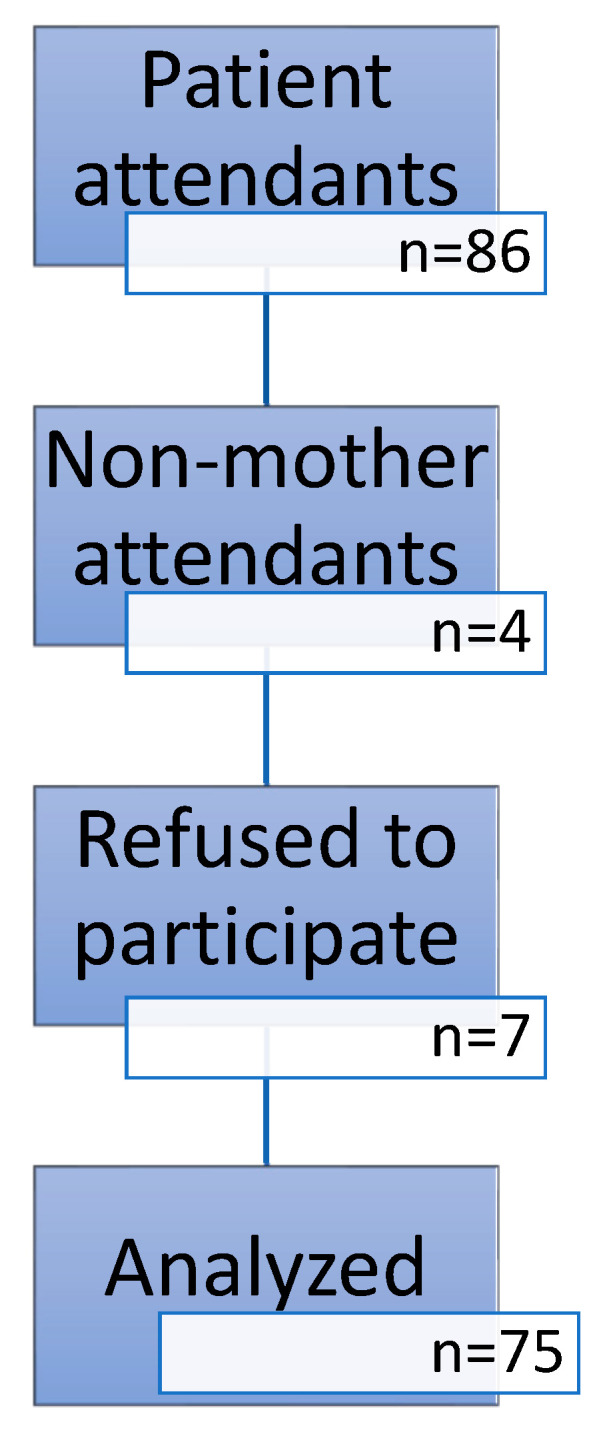
Study flow diagram.

Since the inclusion of all mothers during the study period was targeted, no sample size was calculated. There were a total of 86 patient attendants, 4 being not the mother of the hospitalized patient. Seven mothers refused to participate in the study (Figure 1).

### 2.5. Variables

Data collection was carried out through a face-to-face interview method by experienced nurses working in pediatric clinics using a sociodemographic data form, the Beck Anxiety Inventory (BAI), the Beck Depression Inventory (BDI), the Fear of COVID-19 Inventory, and the Coronavirus Anxiety Inventory. The BAI was developed by Beck et al. in 1988 and validated in Turkish by Ulusoy et al. in 1998 [15,16]. The BDI was developed by Beck in 1961 and validated in Turkish by Hisli N in 1989 [17,18]. The Fear of COVID-19 Inventory was developed by Ahorsu in 2020 and validated in Turkish by Ladikli in 2020 [19,20]. The Coronavirus Anxiety Inventory was developed by Lee in 2020 and validated in Turkish by Biçer in 2020 [21,22]. The primary variable of the study was having anxiety. A BAI score of <8 was interpreted as “no anxiety”, 8–15 as “mild”, 16–25 as “moderate”, and 26–63 as “severe”. A BDI score of <9 was inferred as “minimal”, 10–16 as “mild”, and ≥17 as “moderate–severe”. Other independent variables were the duration of the stay in the ICU, the mothers’ age, educational status, location (urban/district/rural), work status, and COVID-19 history.

### 2.6. Statistical Analysis

Statistical analysis was performed via the Statistical Package for the Social Sciences (SPSS) (SPSS for Windows, Version 25.0, Chicago, IL, USA) program. Numerical variables were presented as the median and interquartile range (IQR), while categorical variables were presented as frequency and percentage. Normal distribution was evaluated via the Kolmogorov–Smirnov test. Numerical data were compared between groups using the Kruskal–Wallis or Mann–Whitney U test. The chi-square (or Fisher’s exact) test was used to compare categorical variables. Logistic regression analysis was used to check for factors independently affecting the presence of anxiety. A *p*-value of <0.05 was considered sufficient for statistical significance.

Informed consent was obtained from all of the participants in this study.

## 3. Results

The median age of the participants was 33 (min: 21, max: 50). Eighty-eight percent of the mothers were not working. The most common reasons for admission to the PICU were infections (Table 1). Diagnoses other than infections were found to be convulsions, drug intoxications, and other diseases (trauma, metabolic diseases, endocrine diseases, cardiological reasons, and gastrointestinal diseases). Our study did not include patients or mothers with a diagnosis of COVID-19.

The mothers’ Beck Anxiety and Beck Depression scores differed significantly depending on the children’s diagnoses (Table 2).

Additionally, having a relative with a history of COVID-19 was a significant factor affecting the Beck Depression and Coronavirus Anxiety scores. However, it did not have any relationship with the Beck Anxiety and Fear of COVID-19 scores. In addition, the Beck Anxiety scores were significantly higher in mothers with a history of COVID-19 compared to those who did not have the disease. However, work status only had a significant relationship with the Coronavirus Anxiety scale scores. On the other hand, no significant difference was found between the educational groups and their scale scores (Table 2).

Mothers’ Beck Anxiety and Beck Depression scores were significantly different according to the children’s diagnoses, but the difference in the Beck Anxiety scores was not significant after the Bonferroni correction (Table 3).

Maternal and child ages were significantly higher in participants with anxiety (Table 4). Subgroup comparisons were made according to the presence of anxiety, demonstrating that educational status, personal history of COVID-19, or having a relative with a history of COVID-19 caused statistically significant differences.

The logistic regression model was created including variables that were significant in pairwise comparisons. The model had a Nagelkerke R square value of 0.534 with a 66.7% sensitivity and 96.7% specificity in classifying the anxiety status of mothers. In this model, a previous experience of COVID-19 affected anxiety independently. A history of COVID-19 increased the risk of developing anxiety by approximately 15 times (Table 5).

## 4. Discussion

To our knowledge, this is the first study to evaluate the depression and anxiety of mothers of children hospitalized in the pediatric ICU during the COVID-19 pandemic. The rate of depressive symptoms (45%) in mothers of children hospitalized in the intensive care unit during this period showed how important the issue was. Especially in some specific situations, mothers may be more prone to depressive symptoms. Similarly to our study, the mental health of mothers deteriorated in studies conducted on children or those in need of special attention and education during the COVID-19 pandemic [1,23].

Compared with adults, children with COVID-19 have milder symptoms and a milder disease course. However, children with underlying medical problems and infants under 1 year of age are at an increased risk of disease severity [24]. These factors may have adversely affected the mental health of parents with children in need of intensive care.

The unusual environment with strange equipment in the PICU, bright lights, smells, insomnia, parents seeing invasive attempts on their child, and the presence of a serious medical event that threatens their child’s life are sources of stress for parents [25]. Additionally, situations such as fear of infection, economic pressures, rapid changes in lifestyle, and the closure of schools in the COVID-19 pandemic are also important stress factors for parents’ mental health [26,27]. Moreover, the risk of developing depressive symptoms is higher in those exposed to such stressors [28].

In the COVID-19 pandemic, anxiety symptoms increased in patients due to both socio-economic and disease-related reasons [29]. Patients infected with the SARS-CoV-2 virus experienced stress due to social isolation, having a new and potentially deadly infectious disease, and the fear of stigma [29,30]. While most individuals can successfully cope with these stressors, some people may develop mental disorders such as post-traumatic stress disorder, depression, and anxiety. In a systematic review and meta-analysis, caregivers who struggled against the pandemic were found to have high levels of depression [31]. Therefore, apart from all of the factors affecting the psychological health of mothers, it is also necessary to consider the burden of experiencing the unusual environment of hospitals during the pandemic on the mental health of mothers.

It was expected that the anxiety of the relatives of the patients would increase in the intensive care environment. However, it was surprising that the anxiety levels of mothers with COVID-19 were 15 times higher than those without.

In a study of 402 patients with COVID-19, 42% of participants had anxiety symptoms [29]. In another study, severe anxiety symptoms were detected in all patients hospitalized for COVID-19 [32]. The findings of our research support these studies. This can be explained by the fact that inflammatory processes cause psychiatric symptoms [33], or it can be attributed to reasons such as quarantine, fear of death, and fear of losing relatives [34].

Anxiety and depression levels are high in parents of children with neurological or neurodevelopmental disorders [35]. Similarly, in this study, the parents of the patients who were hospitalized in the intensive care unit due to convulsions were more depressed than the other parents. It should not be ignored that mothers of children hospitalized in the PICU after convulsions may need psychiatric support.

In our study, the mothers’ anxiety levels increased with increasing maternal and child age. We could not find any study in the literature investigating this relationship. However, it has been shown that anxiety symptoms occur as the child’s age increases, depending on the anxiety levels of the mothers in the early childhood period [36]. Anxiety may occur in children in the intensive care unit whose mothers have anxiety symptoms due to exposure to these signs. On the other hand, the increasing awareness of children in the intensive care unit, which is a strange or frightening environment, as they get older, and the development of their expressive skills may cause an increase in their mothers’ anxiety levels. This dual effect can be clarified by future studies.

It is a new finding that mothers with a family history of COVID-19 show lower levels of depressive symptoms. No study was found in the literature on these data. However, stress increases the symptoms of depression [37]. Anxiety about the consequences can cause depression in people who have not had COVID-19. Perhaps the anxiety of people who have had COVID-19 in their relatives is decreased by the disappearance of uncertainty.

Reasons such as the risk of contamination in the workplace, the possibility of being quarantined, and complications that may develop in case of possible contamination may have caused the Coronavirus Anxiety levels to be higher in working mothers than in non-working mothers.

## 5. Study Limitations

In this article, it is worth mentioning some of the limitations of the study. Factors that may affect the psychiatric conditions and anxiety levels of other family members have not been investigated. The questionnaires were not repeated after the patients were discharged. It was not determined whether there was a change in the anxiety levels of the mothers. In addition, the lack of representativeness, the fact that only one clinic was included, and that the study is only cross-sectional with one point in time of data collection may have affected the analysis results in statistical comparisons.

## 6. Conclusions

The COVID-19 pandemic is a crisis that undermines the social and economic order and established values, and causes uncertainty, fear, and anxiety. Considering that there may be psychological consequences such as shock, denial, anxiety, worry, and stress caused by the COVID-19 pandemic, high-risk groups such as children, the elderly, women, healthcare workers, people with long-term hospitalizations, and their relatives should be prioritized. It is important to work on crisis and stress management, awareness- and compassion-based activities, coping resources, and strengthening social support resources for these groups.

When children of mothers with a history of COVID-19 are hospitalized, special attention should be given to their mothers concerning psychological support and assistance. Relatives of children with certain diagnoses may be at a higher risk of anxiety about new, contagious, and poorly understood diseases such as COVID-19.

## Figures and Tables

**Table 1:** Descriptive features of the participants.

		n	%
Diagnosis in childhood	Convulsion	20	26.7
	Drug intoxication	14	18.7
	Infections	23	30.7
	Other	18	24.0
Education	Primary school	14	18.7
	Secondary school	39	52.0
	High school	10	13.3
	University	12	16.0
Location	Village/rural area	18	24.0
	District	21	28.0
	City center/urban area	36	48.0
Work	Working	9	12.0
	Not working	66	88.0
COVID-19 history	Yes	16	21.3
	No	59	78.7

**Table 2:** Comparison of scale scores according to descriptive characteristics.

	Med	25–75%	Med	25–75%	Med	25–75%	Med	25–75%		
Diagnosis	Convulsion		Drug Intoxication		Infections		Other		H	P
Beck Anxiety score	1	0–3	5	1–8	4	2–7	6	5–13	11.222	0.011
Beck Depression score	13	2–16	6	1–12	10	6–13	6	3–9	8.698	0.034
Coronavirus Anxiety score	0	0–1	0	0–1	0	0–0	0	0–0	3.892	0.273
Fear of COVID-19 score	15	14–21	13	8–18	13	9–25	20	11–23	4.110	0.250
Education	Primary		Secondary		High school		University		H	P
Beck Anxiety score	7	0–13	4	2–7	3	0–8	2	1–7	1.829	0.609
Beck Depression score	6	6–9	9	2–15	12	10–13	10	5–12	5.203	0.158
Coronavirus Anxiety score	0	0–0	0	0–1	0	0–1	0	0–0	1.804	0.614
Fear of COVID-19 score	13	7–22	15	11–25	14	14–19	16	9–24	1.837	0.607
Location	Village		District		City				H	P
Beck Anxiety score	7 _a_	2–13	2 _b_	0–6	3 _b_	0–7			8.002	0.018
Beck Depression score	10	5–13	9	3–10	10	4–12			0.562	0.755
Coronavirus Anxiety score	0	0–3	0	0–0	0	0–1			0.949	0.622
Fear of COVID-19 score	16	12–25	11	8–18	16	12–22			3.291	0.193
Having a relative had COVID-19	Yes		No						Z	P
Beck Anxiety score	4	2–8	4	1–7					0.804	0.422
Beck Depression score	6	3–10	10	5–15					2.088	0.037
Coronavirus Anxiety score	0	0–0	0	0–1					2.050	0.040
Fear of COVID-19 score	17	8–26	14	11–22					0.332	0.740
Did she have COVID-19?	Yes		No						Z	P
Beck Anxiety score	7	4–13	2	0–7					3.092	0.002
Beck Depression score	6	3–15	9	4–13					0.026	0.979
Coronavirus Anxiety score	0	0–11	0	0–0					1.313	0.189
Fear of COVID-19 score	13	7–28	15	11–22					0.291	0.771
Working	Yes		No						Z	P
Beck Anxiety score	1	0–4	4	2–7					1.154	0.248
Beck Depression score	6	5–31	9	3–13					1.055	0.291
Coronavirus Anxiety score	1	0–3	0	0–0					2.788	0.005
Fear of COVID-19 score	18	11–20	14	10–22					0.131	0.896

H: Kruskal–Wallis test value, Z: Mann–Whitney U test value, Med: median. Each subscript letter denotes a subset of Beck Anxiety scores whose column medians do not differ significantly from each other at the 0.05 level.

**Table 3:** Comparison of scale degrees regarding diagnosis.

		Diagnosis									
		Convulsion		Drug Intoxication		Infections		Other			
		n	%	n	%	n	%	n	%	χ^2^	P
Beck Anxiety evaluation	No anxiety	17 _a_	85.0	10 _a_	71.4	21 _a_	91.3	12 _a_	66.7	13.951	0.022
	Mild	1 _a_	5.0	2 _a_	14.3	2 _a_	8.7	6 _a_	33.3		
	Moderate	0 _a_	0.0	2 _a_	14.3	0 _a_	0.0	0 _a_	0.0		
	Severe	2 _a_	10.0	0 _a_	0.0	0 _a_	0.0	0 _a_	0.0		
Beck Depression evaluation	Minimal	6 _a_	30.0	8 _a.b_	57.1	10 _a_	43.5	17 _b_	94.4	23.346	<0.001
	Mild	10 _a_	50.0	4 _a.b_	28.6	13 _a_	56.5	1 _b_	5.6		
	Moderate–severe	4 _a_	20.0	2 _a_	14.3	0 _a_	0.0	0 _a_	0.0		

χ^2^: Fisher’s exact test value. Each subscript letter denotes a subset of “Diagnosis in childhood” categories whose column proportions do not differ significantly from each other at the 0.05 level.

**Table 4:** Comparison of the characteristics of the participants regarding the presence of anxiety.

		Anxiety					
		Absent		Present			
Diagnosis in childhood (n-%)	Convulsion	17	28.3	3	20.0	4.747 ^F^	0.182
	Drug intoxication	10	16.7	4	26.7		
	Infections	21	35.0	2	13.3		
	Other	12	20.0	6	40.0		
Education	Primary school	7 _a_	11.7	7 _b_	46.7	11.461 ^F^	0.005
	Secondary school	36 _a_	60.0	3 _b_	20.0		
	High school	7 _a_	11.7	3 _a_	20.0		
	University	10 _a_	16.7	2 _a_	13.3		
Location (n-%)	Village/rural area	13	21.7	5	33.3	1.142 ^F^	0.612
	District	18	30.0	3	20.0		
	City	29	48.3	7	46.7		
Work (n-%)	Working	7	11.7	2	13.3	0.032 ^F^	1.000
	Not working	53	88.3	13	86.7		
History of COVID-19 (n-%)	Yes	8	13.3	8	53.3	11.441 ^F^	0.002
	No	52	86.7	7	46.7		
Relative with a history of COVID-19 (n-%)	Yes	18	30.0	9	60.0	4.688 ^C^	0.030
	No	42	70.0	6	40.0		
Days spent in the PICU (median–IQR)		2	1–2	2	1–4	1.387 ^Z^	0.166
Maternal age (median–IQR)		31	28–35	40	36–41	4.076 ^Z^	<0.001
Child’s age on admission to the PICU (median–IQR)		27	8–77	152	25–204	3.646 ^Z^	<0.001

^Z^: Mann–Whitney U test, ^F^: Fisher’s exact test, ^C^: chi-square test, IQR: interquartile range, PICU: pediatric intensive care unit. Each subscript letter denotes a subset of Anxiety categories whose column proportions do not differ significantly from each other at the 0.05 level.

**Table 5:** Computer output of the logistic regression model for the presence of anxiety.

	B	Wald	P	Exp (B)	95% CI for EXP (B)	
					Lower	Upper
Mother’s age	0.113	1.397	0.237	1.120	0.928	1.352
History of COVID-19	2.702	7.212	0.007	14.915	2.075	107.192
Age on admission to the PICU	0.009	1.480	0.224	1.009	0.995	1.023
Education (reference category: primary school)		3.399	0.334			
Education (secondary vs. primary)	−1.741	1.917	0.166	0.175	0.015	2.061
Education (high school vs. primary)	0.145	0.012	0.913	1.156	0.086	15.506
Education (university vs primary)	−0.053	0.001	0.972	0.949	0.052	17.283
Has his or her relative had COVID-19?	−0.192	0.036	0.849	0.825	0.115	5.948
Constant	−5.250	2.685	0.101	0.005		

CI: confidence interval.
